# P38 Regulates Kainic Acid-Induced Seizure and Neuronal Firing via Kv4.2 Phosphorylation

**DOI:** 10.3390/ijms21165921

**Published:** 2020-08-18

**Authors:** Jia-hua Hu, Cole Malloy, Dax A. Hoffman

**Affiliations:** Molecular Neurophysiology and Biophysics Section, The Eunice Kennedy Shriver National Institute of Child Health and Human Development, Bethesda, MD 20892, USA; jia-hua.hu@nih.gov (J.-h.H.); cole.malloy@nih.gov (C.M.)

**Keywords:** Kv4.2, seizure, p38 MAPK, temporal lobe epilepsy, hippocampus, neuronal firing and excitability

## Abstract

The subthreshold, transient A-type K^+^ current is a vital regulator of the excitability of neurons throughout the brain. In mammalian hippocampal pyramidal neurons, this current is carried primarily by ion channels comprising Kv4.2 α-subunits. These channels occupy the somatodendritic domains of these principle excitatory neurons and thus regulate membrane voltage relevant to the input–output efficacy of these cells. Owing to their robust control of membrane excitability and ubiquitous expression in the hippocampus, their dysfunction can alter network stability in a manner that manifests in recurrent seizures. Indeed, growing evidence implicates these channels in intractable epilepsies of the temporal lobe, which underscores the importance of determining the molecular mechanisms underlying their regulation and contribution to pathologies. Here, we describe the role of p38 kinase phosphorylation of a C-terminal motif in Kv4.2 in modulating hippocampal neuronal excitability and behavioral seizure strength. Using a combination of biochemical, single-cell electrophysiology, and in vivo seizure techniques, we show that kainic acid-induced seizure induces p38-mediated phosphorylation of Thr607 in Kv4.2 in a time-dependent manner. The pharmacological and genetic disruption of this process reduces neuronal excitability and dampens seizure intensity, illuminating a cellular cascade that may be targeted for therapeutic intervention to mitigate seizure intensity and progression.

## 1. Introduction

Seizures are a common manifestation of a host of neurological disorders, including epilepsy. Affecting an estimated 1% of the US population [1], these events are characterized by the anomalous synchronization of electrical activity in the brain due to the hyperexcitability of individual neurons and neural networks. Although relatively indiscriminate in their anatomical localization, seizures often occur in the hippocampus and surrounding cortical areas of the temporal lobe. Recurrent seizures in this brain region underlie temporal lobe epilepsy (TLE), which is thought to be the most common epilepsy syndrome in adults [2]. In addition to being a prevalent form of epilepsy, TLE is frequently the most difficult to treat [3]. These seizures are often resistant to antiepileptic drugs, which makes the affected region susceptible to ongoing, intractable epilepsy [4,5,6]. For up to one-third of patients, surgical intervention is encouraged for mitigation [1,7,8]. As a result, an emphasis on the identification of additional mechanisms underlying TLE, which can serve as potential targets for therapy, is requisite.

While the etiology of seizures underlying TLE is multifaceted, a common feature is the dysfunction of voltage-gated ion channels in hippocampal pyramidal neurons. A host of channelopathies—both inherited and acquired—are associated with TLE in this principal cell type, including in hyperpolarization-activated cyclic nucleotide-gated (HCN) channels [9,10,11,12,13], voltage-gated Na^+^ channels (Na_v_) [14,15], and voltage-gated Ca^2+^ channels (Ca_v_) [16,17]. Featured most prominently in TLE pathologies are abnormalities in the function of voltage-gate K^+^ channels (K_v_), which are critical regulators of the intrinsic excitability of neurons throughout the brain (reviewed in [18,19]). Although members of all classes of K^+^ channels have been shown to be altered in various epilepsy syndromes [19], there has been a steady increase in findings linking A-type K^+^ channels (Shal subfamily) to TLE. Chief among the members of this family is Kv4.2, which has been heavily implicated in TLE in both animal models [19] and humans [20,21,22]. Kv4.2 is the primary pore-forming K_v_ channel subunit underlying the rapidly activating and inactivating somatodendritic A-current (I_A_) in CA1 pyramidal neurons of the hippocampus [23,24,25]. Operating at subthreshold voltages, Kv4.2 regulates action potential (AP) repolarization and repetitive firing, dampens AP backpropagation into dendrites, and shapes synaptic potentials, thus acting as a powerful modulator of the input–output efficacy of pyramidal neurons [23,26,27]. Although mutations in the Kv4.2 gene that impart defects intrinsic to channel function are associated with TLE [20,21,22,23], it is also evident that disruptions/modifications in Kv4.2 channel properties occur *in response* to seizures, suggesting that regulation of these channels likely contributes to the intractability of TLE. Indeed, TLE has been shown to decrease Kv4.2 availability [28,29], however, the molecular mechanisms underlying this activity-induced downregulation remain unclear.

Substantial evidence supports the notion that Kv4.2 channels function in macromolecular complexes with auxiliary subunits, including the K^+^ channel-interacting proteins (KChIP1-4) and dipeptidyl peptidases 6 and 10 (DPP6 and DPP10) [30]. Both KChIPs and DPPs work together to exert the strong modification of Kv4.2 expression, membrane surface localization, and channel kinetics [31,32,33,34]. Evidence of increased seizure susceptibility is present in mice harboring mutations in these auxiliary subunits, including KChIP 2 [35], suggesting the maintenance of channel complexes is a key factor in moderating seizures. Likely modulators of Kv4.2 complex dynamics are protein kinases. The phosphorylation of Kv4.2 by protein kinase A (PKA), protein kinase C (PKC), and extracellular signal-regulated kinase/mitogen-activated protein kinase (ERK/MAPK) downregulates I_A_ [36,37,38]. In pyramidal neurons, this downregulation facilitates an increase in somatodendritic excitability, enhancing susceptibility to network hyperexcitability in the hippocampus [39,40,41]. We have recently identified a specific MAPK, p38α, as a potent regulator of the Kv4.2 complex [42]. The p38 phosphorylation of Kv4.2 C-terminal motifs triggers a molecular cascade that facilitates the dissociation of Kv4.2 from its auxiliary subunit DPP6 [42]. This cascade is particularly intriguing in the context of TLE, as it occurs in an activity-dependent fashion, representing a novel mechanism that may be integral in regulating seizure susceptibility [42].

In the present study, we expand on our previous findings and address how p38 kinase modulates seizure susceptibility and neuronal excitability. We use biochemical, electrophysiological, and in vivo seizure techniques in WT and a novel mouse model harboring a point mutation preventing p38 phosphorylation of Kv4.2 at C-terminal Thr607 (Kv4.2TA) to illuminate the role of p38 phosphorylation of Kv4.2 in regulating the intrinsic excitability of hippocampal pyramidal neurons and seizure intensity. We show that p38 phosphorylation of Kv4.2 at C-terminal Thr607 is integral in modulating seizure strength and may contribute to the progression of seizure intensity over time. Furthermore, we confirm previous findings that a molecular cascade triggered by p38 phosphorylation alters Kv4.2-mediated excitability of hippocampal pyramidal neurons, illuminating a novel molecular mechanism involved in network hyperexcitability in mice. The combined pharmacological and genetic manipulation of the cellular cascade described here offers insight into various avenues through which therapeutic intervention to curtail seizure progression can be pursued.

## 2. Results

### 2.1. p38 MAPK Contributes to Kainic Acid-Induced Seizure in WT but Not Kv4.2TA Mice

**Figure 1:**
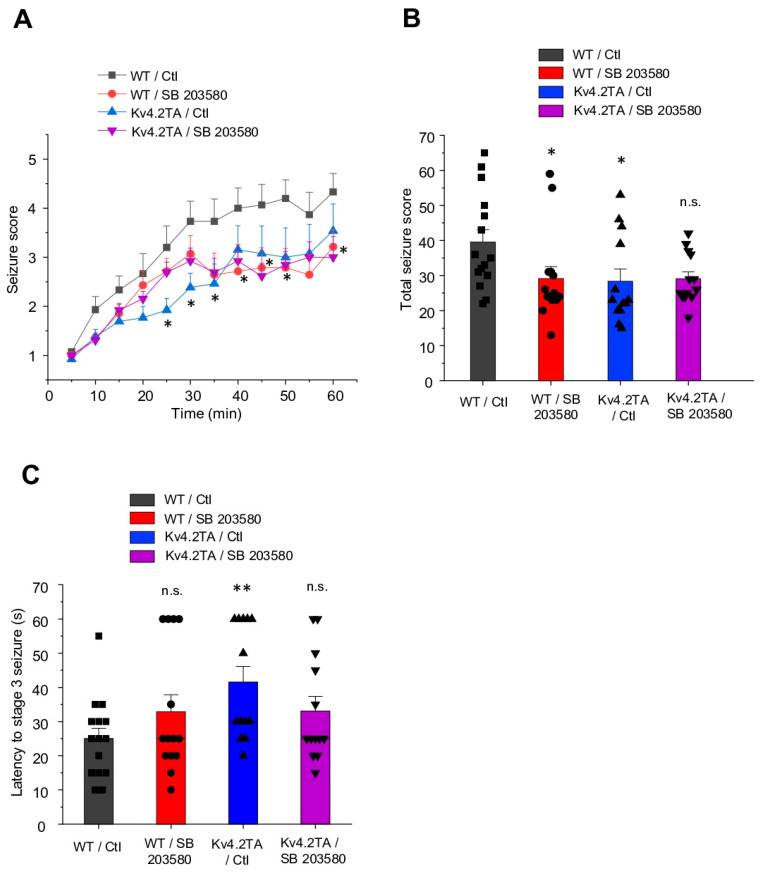
p38 mitogen-activated protein kinase (MAPK) contributed to kainic acid-induced seizure in WT mice but not Kv4.2TA mice. (**A**) Time course of mean behavioral seizure score following kainic acid injection. The mean behavioral seizure score was significantly reduced in Kv4.2TA mice compared to WT mice. Furthermore, p38 inhibitor SB 203580 significantly reduced behavioral seizure score following kainic acid injection in WT mice but not in Kv4.2TA mice, *n* = 13–15 for each group, two-way ANOVA, * *p* < 0.05. (**B**) Total behavioral seizure score for each group, *n* = 13–15 for each group, *t*-test, * *p* < 0.05. (**C**) Latency to stage 3 seizure for each group. *n* = 13–15 for each group, *t*-test, ** *p* < 0.01.

We have generated a mutant mouse, Kv4.2TA, with abolished dynamic Thr 607 phosphorylation of Kv4.2 and isomerization of Kv4.2 by Pin1 [42]. Kv4.2TA mice displayed increased I_A_, decreased neuronal excitability, and improved cognitive flexibility [42]. Here, we examine if acute behavioral seizure is altered following the systemic injection of kainic acid (KA) in the Kv4.2TA mice. KA (25 mg/kg) was injected intraperitoneally into Kv4.2TA mice (*n* = 13) and littermate controls (*n* = 15) and behavioral seizure responses were scored using the modified Racine scale [43] for 60 min post injection. We observed a significant difference in behavior seizure scores, with Kv4.2TA mice showing significantly reduced seizure intensity over the full 1 h period following KA injection (Figure 1A,B). Our previous work showed that p38 can phosphorylate Kv4.2 at T607 in response to seizure induced by pentylenetetrazol (PTZ) or KA [42]. In addition, acute behavioral seizure in p38 knockout mice is significantly decreased compared to WT mice [44]. Therefore, we hypothesized that the effect of p38 on seizure intensity is dependent on the T607 site of Kv4.2. We injected p38 inhibitor SB 203580 (20 mg/kg, i.p.) 15 min ahead of KA injection. The result showed that control mice with SB 203580 injection (*n* = 14) exhibited significantly reduced behavioral seizure intensity compared to those with the control injection (Figure 1A,B). Interestingly, Kv4.2TA mice with SB 203580 injection (*n* = 13) did not display a significant reduction in behavioral seizure intensity compared to those injected with vehicle (Figure 1A,B). This decrease in sensitivity to KA-induced seizure was also reflected in the latency to stage 3 seizure (Figure 1C). Taken together, these data support the notion that p38 phosphorylation of Kv4.2 at T607 contributes to KA-induced seizure.

### 2.2. Seizure Induced by Kainic Acid Triggers Kv4.2 T607 Phosphorylation in a Time-Dependent Manner

**Figure 2:**
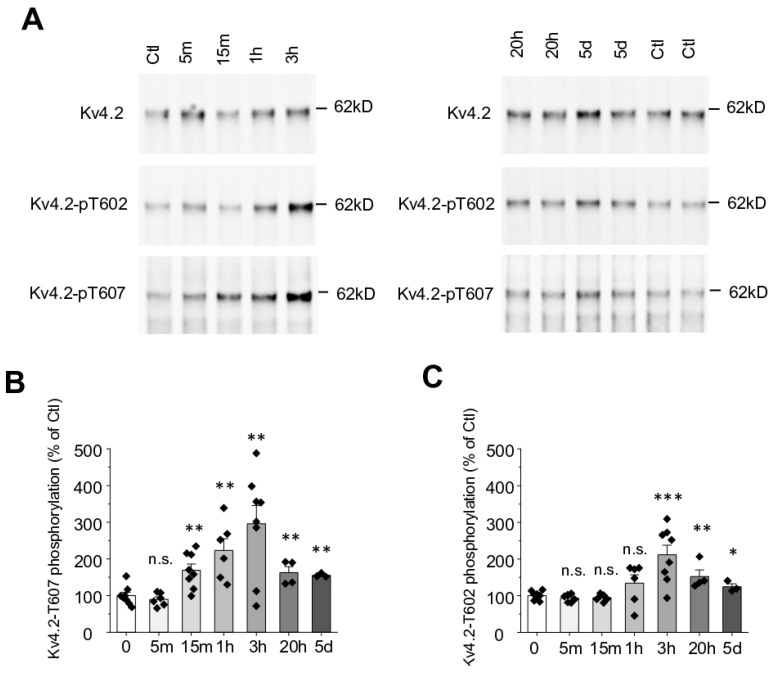
Seizure induced by kainic acid triggers Kv4.2 T607 phosphorylation in a time-dependent manner in mouse hippocampus. (**A**) Time course of Kv4.2 phosphorylation at Thr602 and Thr607 by kainic acid administration (25 mg/kg, i.p.) in mouse hippocampus. (**B**) Statistical analysis of kainic acid-induced phosphorylation of Kv4.2 at Thr607 in mouse hippocampus, *n* = 3–8 in each group, *t*-test, ** *p* < 0.01. (**C**) Statistical analysis of kainic acid-induced phosphorylation of Kv4.2 at Thr602 in mouse hippocampus, *n* = 3–8 in each group, *t*-test, * *p* < 0.05, ** *p* < 0.01, *** *p* < 0.001.

It has been reported that Kv4.2 availability is altered in response to seizure, suggesting these events may trigger a molecular cascade leading to the functional downregulation of I_A_ [28,29]. Our collective analyses indicate that this cascade is initiated by p38 kinase. We reason that the prolonged activation of p38 and subsequent phosphorylation of Kv4.2 in response to continued seizures (kindling) may be an important factor underlying the intractability of seizures in the temporal lobe. Therefore, in order to study the timing of Kv4.2 phosphorylation in response to seizure, we examined Kv4.2 phosphorylation at various times following KA injection. Since p38 can phosphorylate both T602 and T607 of Kv4.2 [42], we examined both phosphorylation sites. KA induced Kv4.2 phosphorylation at T607 at 15 min after KA injection but not at 5 min (Figure 2A,B). T607 phosphorylation peaked 3 h after KA injection and the induction effect lasted, even at 5 days post injection (Figure 2A,B). Kv4.2 phosphorylation at T602 was also induced 3 h post KA injection but not at earlier time points (Figure 2A,C). These data show that seizure induced by KA triggers a long-lasting effect on Kv4.2 that may contribute to on-going seizure progression.

### 2.3. Kainic Acid-Induced Kv4.2 Phosphorylation at T607 Is Dependent on p38 MAPK

**Figure 3:**
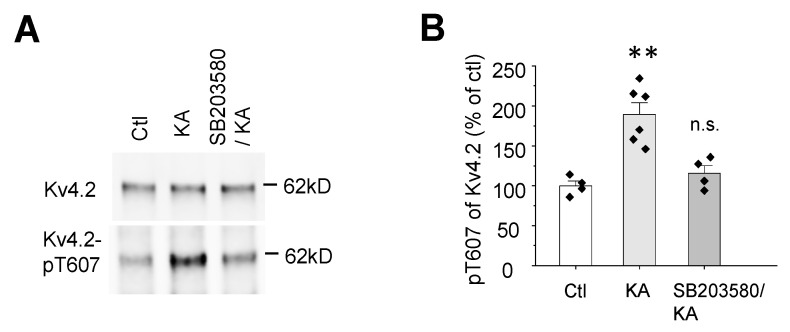
p38 MAPK contributes to kainic acid-induced Kv4.2 phosphorylation at T607. (**A**) SB 203580, a potent p38 inhibitor (20 mg/kg, i.p., 15 min), blocked kainic acid-induced phosphorylation of Kv4.2 T607 in mouse hippocampus. (**B**) Statistical analysis of the effect of SB 203580 on kainic acid-induced phosphorylation of Kv4.2 at Thr607 in mouse hippocampus, *n* = 4–6 in each group, *t*-test, ** *p* < 0.01.

Next, we wanted to know if p38 is required for KA-induced Kv4.2 phosphorylation at T607. The p38 inhibitor SB 203580 (20 mg/kg, i.p.) was injected 15 min ahead of KA injection (25 mg/kg, i.p.). We found that the p38 inhibitor SB 203580 blocked the induction of Kv4.2 phosphorylation 15 min after KA injection in the mouse hippocampus (Figure 3A,B), suggesting p38 contributed to KA-induced Kv4.2 phosphorylation at T607.

### 2.4. p38 MAPK Colocalizes with Kv4.2

**Figure 4:**
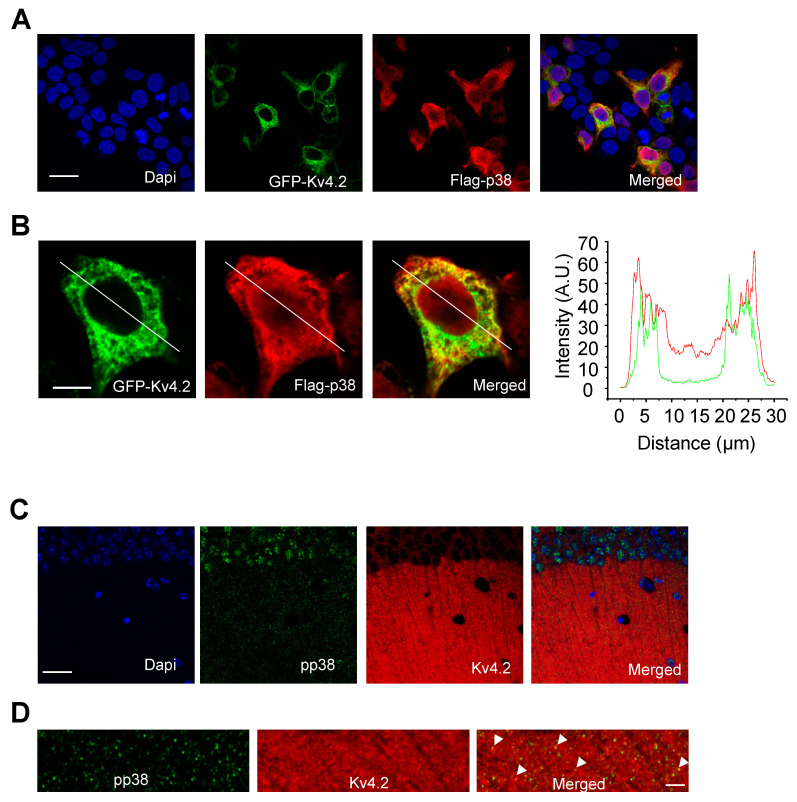
p38 MAPK colocalizes with Kv4.2. (**A**) HEK293T cells were transfected with GFP-Kv4.2 and Flag-p38. Cells were fixed and stained with GFP and Flag to show co-localization. Scale bar: 20 μm. (**B**) High magnification images and line scan analysis of colocalization. Scale bar: 5 μm. (**C**) Mouse brains were co-stained with Kv4.2 and pp38 antibody. Phosphorylated p38 is localized in the cell body and dendrites as well. Scale bar: 20 μm. (**D**) High magnification images showing Kv4.2 and pp38 colocalized in dendrites, as indicated with arrow heads. Scale bar: 5 μm.

Since p38 phosphorylates Kv4.2, we wanted to see if it colocalized with Kv4.2 in a heterologous system and in the mouse brain. First, HEK 293T cells were double stained after 2 days of transfection with p38 and Kv4.2. The result showed that p38 partially colocalized with Kv4.2 (Figure 4A). High magnification images and line scan confirmed this result (Figure 4B,C). In addition, we performed the double staining of phosphorylated p38 (pp38) and Kv4.2 on mouse brain sections. Phospho-p38 is mainly localized in the cell body of hippocampal pyramidal neurons but also localized in dendrites, while Kv4.2 is mainly localized in dendrites (Figure 4C). High magnification images showed pp38 partially colocalized with Kv4.2, as indicated by arrow heads (Figure 4D).

### 2.5. Kainic Acid Activates p38 MAPK in both WT and Kv4.2TA Mice

**Figure 5:**
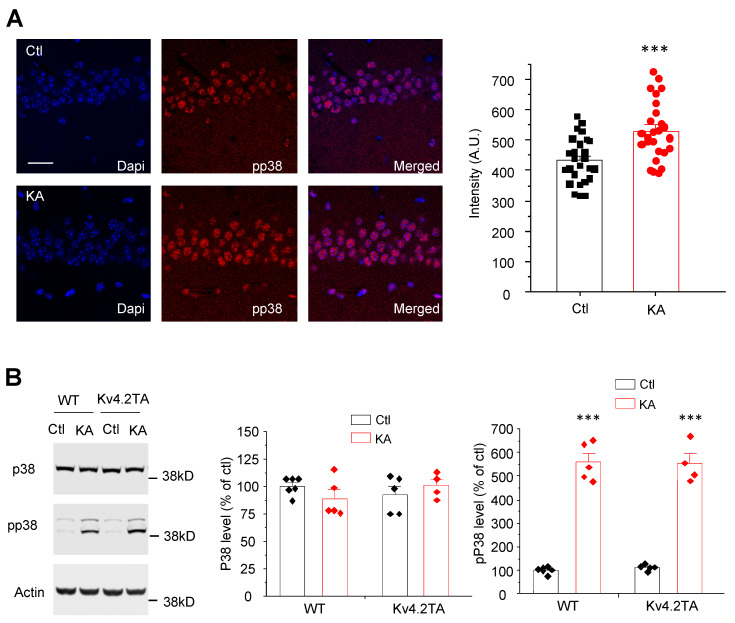
Kainic acid activates p38 MAPK in both WT and Kv4.2TA mice. (**A**) Immunostaining analysis showed p38 phosphorylation increased with kainic acid administration (25 mg/kg, i.p., 30 min) in mouse hippocampus, *n* = 26 cells in each group, *t*-test, *** *p* < 0.001. (**B**) Western blot analysis showed p38 phosphorylation increased with kainic acid administration (25 mg/kg, i.p., 30 min) in hippocampus in both WT and Kv4.2TA mice, *n* = 4–6 cells in each group, *t*-test, *** *p* < 0.001.

We next assessed whether KA-induced seizure activated p38 in both WT mice and Kv4.2TA mice. Mouse brain sections were stained with pp38. The pp38 level is significantly increased after KA injection (1 h) in the cell body of hippocampal pyramidal neurons (Figure 5A). Furthermore, we examined pp38 by western blot. The pp38 level but not p38 level is significantly increased after KA injection (30 min) in WT mouse hippocampus (Figure 5B). A similar result was found in Kv4.2TA mice (Figure 5B). These data indicate that the initiation of p38 kinase activity by KA is similar in WT and Kv4.2TA mice, and the observed effects on seizure intensity can be ascribed to the inability of p38 to phosphorylate Thr607.

### 2.6. p38 MAPK Modulates Neuronal Excitability through Kv4.2

**Figure 6:**
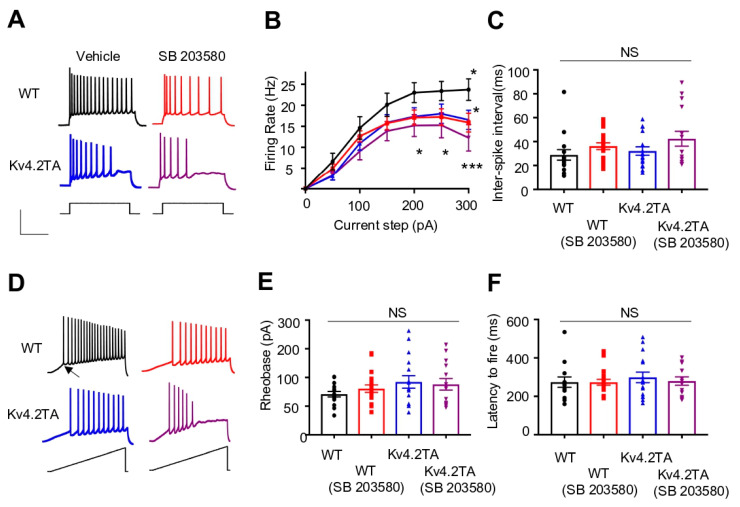
p38 impacts hippocampal pyramidal neuron excitability through Kv4.2. (**A**) Current step of +300 pA induces repetitive firing in pyramidal neurons recorded from WT and Kv4.2TA mice with or without SB 203580 treatment. Scale 40 mV/250 ms. Square current inset 300 pA. (**B**) Sequential somatic current injections increasing in magnitude reveal p38 kinase inhibition reduces AP firing frequency in WT hippocampal neurons at +300 pA relative to vehicle (*n* = 15 in vehicle, *n* = 19 in treatment; two-way ANOVA, * *p* < 0.05). Kv4.2TA neurons display reduced firing frequency at +300 pA relative to WT in vehicle, which is augmented in the presence of SB 203580 such that current magnitudes of +200 and +250 pA also exhibit significant differences (*n* = 18 in vehicle, *n* = 14 in SB 203580; two-way ANOVA, * *p* < 0.05; *** *p* < 0.001). (**C**) Inter-spike intervals measured between the first two spikes in a train evoked by 150 pA injection display no significant difference among groups. Kruskal–Wallis test, *p* > 0.05. (**D**) Ramp current injections evoke repetitive firing in all pyramidal neurons recorded in each condition. Arrow indicates point at which action potential (AP) threshold, rheobase, and latency to fire were measured. Ramp current inset 400 pA/s. (**E**) Minimum current to elicit AP firing at threshold (rheobase) is not significantly different among the populations. One-way ANOVA, *p* > 0.05. (**F**) Latency to fire in response to ramp injection is not significantly different among populations. Kruskal–Wallis test, *p* > 0.05.

In our previous study, we reported that hippocampal pyramidal cells from acute hippocampal slices of Kv4.2TA mice exhibited a nearly two-fold reduction in AP firing frequency in response to somatic current injection relative to WT mice. We determined that this was due to an enhancement of I_A_ as a result of the T607A mutation blocking the dissociation of Kv4.2 from its auxiliary subunit DPP6 and subsequent functional downregulation [42]. Furthermore, we found the slicing process largely activated p38 kinase and induced Kv4.2 phosphorylation, revealing that this procedure acts similarly to kainic acid-induced seizure in altering the phospho-state of Kv4.2 in the hippocampus [42]. Therefore, we sought to investigate how a pharmacological blockade of p38 (SB 203580) altered the excitability of the principal hippocampal neurons in area CA1 of acute hippocampal slices. In light of the time-dependency of phosphorylation of Kv4.2 at T602 and T607 in response to seizure induction (peak ~3 h), we incubated slices in recovery solution with pharmacological treatment or vehicle (0.1% DMSO) for 2 h and continued their exposure during electrophysiological recordings (3–4 h total exposure). In response to stepped somatic current injections, we identified that treatment with 5 µM SB 203580 reduced AP firing frequency of pyramidal neurons at each magnitude above rheobase in WT slices (Figure 6A,B). At maximum current injection (+300 pA), the difference in firing frequency reached a statistically significant level. Specifically, a +300 pA injection induced a firing rate of 23.7 Hz in the presence of DMSO, representing a~33% increase relative to the rate recorded in the presence of 5 µM SB 203580 (16.9 Hz), shown in Figure 6B. This alteration in excitability was limited to suprathreshold properties, as subthreshold excitability was largely unaffected by pharmacological p38 blockade (Table 1). Parameters measured from ramp current injections (400 pA/s), including AP threshold (Table 1), rheobase (Figure 6D,E), and latency to AP onset (Figure 6F), were similar in conditions with or without SB 203580, although a modest, but non-statistically significant, increase in AP threshold and rheobase was observed in slices from WT mice with pharmacological p38 blockade (Table 1 and Figure 6E).

We next tested the effect of SB 203580 on slices obtained from Kv4.2TA mice. Because p38-mediated phosphorylation of Kv4.2 is significantly reduced in these mice, we anticipated the mutation would occlude the impact of SB 203580 on neuronal firing if p38 mediates excitability primarily through its regulation of I_A_. Generally consistent with previous observations, pyramidal neurons from Kv4.2TA slices exhibited reduced AP firing frequency relative to WT in multiple experimental conditions (Figure 6A,B). AP firing frequency was reduced at each current magnitude, with a statistically significant reduction exhibited at peak injection in the presence of DMSO (17.5 Hz vs. 23.7 Hz, Kv4.2TA vs. WT, respectively; *p* < 0.05, Figure 6B). The significant decrease in AP firing frequency corresponded with a significant increase in AP threshold in Kv4.2TA neurons relative to WT in this condition (Table 1). Additionally, AP firing mirrored that of WT neurons in the presence of 5 µM SB 203580 (Figure 6B). Importantly, SB 203580 treatment in Kv4.2TA slices did not significantly reduce suprathreshold excitability, contrary to its impact in WT pyramidal neurons (Figure 6B). This suggests that p38 modulation of AP firing can primarily be traced to its regulation of Kv4.2. Furthermore, as noted previously, Kv4.2TA neurons in this condition displayed significant pauses in repetitive firing, which correlated with slight, non-statistically significant, increases in fast after-hyperpolarization amplitudes and inter-spike intervals (Table 1 and Figure 6C, respectively). Therefore, taken together, pharmacological blockade of p38 kinase reduced the suprathreshold excitability of hippocampal pyramidal neurons. The T607A mutation occludes the action of SB 203580, suggesting its impact on AP firing frequency is mediated predominantly through its modulation of Kv4.2-mediated I_A_.

## 3. Discussion

The present study describes a novel mechanism of KA-induced seizure which involves p38-dependent phosphorylation of Kv4.2 at T607. Both T602 and T607 are phosphorylated by KA but the induction timing is different (Figure 2A–C). KA induces T607 phosphorylation relatively promptly (about 15 min) while T602 phosphorylation is relatively delayed (about 3 h). Both T602 and T607 phosphorylation were sustained for at least 5 days, the longest data point measured (Figure 2A–C). Kindling is a commonly used model for the development of seizures and epilepsy in which the duration and behavioral involvement of induced seizures increases after seizures are induced repeatedly [45]. Repeated seizure could boost Kv4.2 phosphorylation levels at T602 and T607, leading to downregulation and enhanced excitability. Therefore, the long-term phosphorylation response to seizure could be a mechanism of kindling.

We have found that Pin1 binds to T607 of Kv4.2 and isomerizes the T607-P bond to modulate the function of Kv4.2 [42]. The dual phosphorylation of T602 and T607 increases the Pin1 binding ability and therefore improves Kv4.2 modulation. KA triggers Kv4.2 phosphorylation at both sites after 3 h and lasts for 5 days (Figure 2A–C), suggesting that the Pin1 effect could be long lasting as well. The excitability of CA1 pyramidal neuron dendrites was increased in TLE because of the decreased availability of A-type potassium ion channels [28]. Pin1’s long-lasting effect could eventually lead to reduced availability of Kv4.2, which fits with the notion that seizure decreases I_A._ Furthermore, the continued phosphorylation at these sites and persistent Pin1 activity in response to prolonged seizure may exacerbate their severity, manifesting as a positive feedback loop, promoting further downregulation of I_A_. Thus, the activation of p38 and subsequent isomerization of Kv4.2 may serve as a mechanism underlying the intractability of seizure progression often associated with severe epilepsy in the temporal lobe [1,2,3].

Kv4.2 T602 and T607 phosphorylation occur via both p38 MAPK (Figure 2A–C) and ERK MAPK [38,46]. However, the p38 inhibitor SB 203,580 blocked Kv4.2 phosphorylation induced by KA (Figure 3A,B), suggesting the primary role of p38 in seizure. Since ERK MAPK can be activated by KA [47], ERK may also have a contributing effect on Kv4.2. In addition, KA activates p38 in Kv4.2TA mice at similar levels as in WT mice, suggesting the reduced seizure phenotype in Kv4.2TA mice (Figure 1) is not because of the differential induction of p38 but the deficiency of Kv4.2 phosphorylation at T607.

At the cellular level, Kv4.2 phosphorylation by p38 at T607 alters the excitability of hippocampal pyramidal neurons of area CA1. The results presented here indicate that the role of p38 in altering membrane excitability is primarily through a reduction in suprathreshold excitability. While Kv4.2 can impact subthreshold properties in pyramidal neurons, including latency to AP onset [48] and rheobase [42], its role in regulating the frequency of repetitive AP firing is well documented [48,49]. Its robust control of membrane potential fluctuations in response to depolarizing currents from resting potential permits a significant functional interaction with voltage-gated Na^+^ channels [48]. Indeed, we identified a significant increase in AP threshold in Kv4.2TA pyramidal neurons. Moreover, a slight increase in after-hyperpolarization amplitude in these cells is likely contributory in delaying the recruitment of Na^+^ channel activation to stepped current injection, which was evidenced in this study and our previous analysis [42]. It is likely that the enhancement of I_A_ amplitude that results with p38-Pin1 blockade reduces the precision of repetitive spiking through the modulation of voltage-gated channels, driving spiking in hippocampal pyramidal neurons.

It is clear that the T607A mutation induces a reduction of pyramidal cell excitability. The trend in reduced excitability of Kv4.2TA neurons was persistent in this analysis, strengthening our previous findings [42]. While slight variations in neuronal responsiveness to current injections of various magnitudes in Kv4.2TA mice relative to WT were observed in this study relative to [42], the overall trend toward a reduction in repetitive firing remained. Slight alterations in the slice recovery and recording conditions are likely to underlie these differences. Furthermore, by complementing the T607A mutation with pharmacological blockade of p38, we uncovered multiple means by which to lower hippocampal pyramidal cell excitability through Kv4.2. The application of SB 203580 altered the firing mode of WT neurons in a manner that mirrored that of Kv4.2TA neurons in its absence. This suggests that p38 regulation of membrane excitability is primarily mediated through Kv4.2. We did, however, note that, although p38 blockade did not significantly alter AP firing properties in Kv4.2TA slices, a reduction in excitability was generally augmented in its presence. This may imply that the inhibition of phosphorylation of both T602 and T607 sites may, together, produce an additive effect in Kv4.2 regulation of membrane properties. As noted, the dual phosphorylation of these motifs provides an environment particularly conducive to binding multiple domains of Pin1 upon p38 phosphorylation, which could facilitate dissociation of Kv4.2 from DPP6 and downregulation [42]. While this likely contributes to the observed phenotype, we cannot, however, rule out the involvement of additional ion channels that may also be impacted by broad pharmacological p38 blockade.

Furthermore, the breadth of this work and that of our previous study focuses on somatic Kv4.2 channel activity. While somatic Kv4 channels are capable of impacting neuronal firing modes, their privileged distribution in dendrites suggests their control of membrane potentials may be more impactful in these domains [23,50,51,52]. It is possible that p38 phosphorylation of Kv4.2 may contribute to the alteration of dendritic excitability by modulating the coupling of synaptic inputs and AP output. Indeed, in the context of the regulation of DPP6-Kv4.2 dynamics, p38 activity in the apical dendrites may be of particular significance. DPP6 knockout mice exhibit a non-uniformity in their manifestation of alteration in membrane excitability, with dendritic excitability being predominantly impacted [53]. Whether p38 phosphorylation of dendritic Kv4.2 channels may impact dendritic function, and further contribute to increased network hyperexcitability and seizure severity in the hippocampus, is a topic of future investigation.

## 4. Materials and Methods

### 4.1. Animals

C57/BL6J wild-type mice were used in this study. Kv4.2TA mice were generated as described before [42]. Mice were group housed in plastic mouse cages with free access to standard rodent chow and water. The colony room was maintained at 22 ± 2 °C with a 12 h: 12 h light: dark cycle. Kv4.2TA mice were backcrossed at least three generations onto C57/Bl6J mice. Age-matched male adult WT and Kv4.2TA were used. All animal procedures were performed in accordance with guidelines approved by the *Eunice Kennedy Shriver* National Institute of Child Health and Human Development Animal Care and Use Committee and in accordance with NIH guidelines (20-042, 3 April 2020).

### 4.2. Expression Constructs

The human Myc-DDK-Kv4.2 construct was purchased from Origene (Rockville, MD, USA, RC215266). The p38 construct was from Addgene (Watertown, MA, USA, 20351).

### 4.3. Chemicals

All chemicals were purchased: KA (Sigma, St. Louis, MO, USA, K0250) and SB 203580 (Tocris, Minneapolis, MN, USA, 1202). For injections, KA was dissolved in saline; SB 203580 was dissolved in DMSO and 10% Tween 80.

### 4.4. Antibodies

Mouse anti-Kv4.2 (NeuroMab, Davis, CA, USA, 75-016) was used at 1:2000 for western blot, 1:200 for immunostaining, rabbit anti-Kv4.2 (Sigma, St. Louis, MO, USA, P0233) was used at 1:2000 for western blot, pT602 (Santa Cruz, Dallas, TX, USA, SC-16983-R) was used at 1:1000 for western blot, pT607 (Santa Cruz, Dallas, TX, USA, SC-22254-R) was used at 1:500 for western blot, and pp38 (Cell Signaling, Danvers, MA, USA, 4511s) at 1:1000 for western blot, 1:100 for immunostaining. Flag (Sigma, St. Louis, MO, USA, F3165) was used at 1:300 for immunostaining, actin (Sigma, St. Louis, MO, USA, A-1978) was used at 1:10,000 for western blot; Alexa Fluor 488 goat anti-mouse (Invitrogen, Carlsbad, CA, USA, A-11029) was used at 1:500; Alexa Fluor 488 goat anti-rabbit (Invitrogen, Carlsbad, CA, USA, A-11034) was used at 1:500; Alexa Fluor 555 goat anti-mouse (Invitrogen, Carlsbad, CA, USA, A-21424) was used at 1:500; Alexa Fluor 555 goat anti-rabbit (Invitrogen, Carlsbad, CA, USA, A-21429) was used at 1:500; Alexa Fluor 680 goat anti-mouse (Invitrogen, Carlsbad, CA, USA, A-21057) was used at 1:10,000; Alexa Fluor 680 goat anti-rabbit (Invitrogen, Carlsbad, CA, USA, A-21076) was used at 1:10,000; IRDye 800CW goat anti-mouse (Licor, Licoln, NE, USA, 926-32210) was used at 1:10,000, IRDye 800CW goat anti-rabbit (Licor, Licoln, NE, USA, 926-32211) was used at 1:10,000.

### 4.5. Cell Culture and Transfection

HEK-293T cells used in biochemistry experiments were obtained from Dr. Paul Worley’s lab. HEK-293T cells were cultured in DMEM medium containing 10% FBS. Transfections were performed with X-tremeGENE 9 (Sigma, St. Louis, MO, USA, XTG9-RO) according to the manufacturer’s specifications. Cells were harvested about 40 h after transfection.

### 4.6. Western Blot and Quantification

Protein samples were mixed with 4x LDS sample buffer (Invitrogen, Carlsbad, CA, USA, NP0007) and 10x sample reducing agent (Invitrogen, Carlsbad, CA, USA, NP0007) to a final concentration of 1x. Samples were loaded on 4%–12% Bis-Tris gradient gel (Invitrogen, Carlsbad, CA, USA, 12-well, NP0322; 15-well, NP0323). The proteins were transferred to an Immobilon-FL PVDF membrane (EMD Millipore, Burlington, MA, USA, IPFL00010). The membrane was blocked with Odyssey blocking buffer (Li-COR, Licoln, NE, USA, 927-40000) for 1 h at room temperature, followed by incubation with primary antibody in PBS overnight at 4 °C. The membrane was then washed with PBST (PBS, pH 7.4 and 0.1% Tween-20) three times and incubated with secondary antibody in PBS for another hour. After three washes with PBS, the membrane was scanned using an Odyssey imaging system (LI-COR, Licoln, NE, USA) according to the manufacturer’s protocol. Quantification of western blots was carried out using the gel analysis function in ImageJ within the linear range of detection, which is determined by using serial dilutions of a representative sample.

### 4.7. Immunostaining

Mice were fixed with 4% PFA and brain sections were cut into 24-well plates. They were then blocked with 10% horse serum at RT for 1 h and then incubated with primary antibodies at 4 °C overnight. After washing, sections were incubated with anti-mouse-555 and anti-rabbit-488 secondary antibodies at RT for 2 h. After washing, cells were then mounted on slides with anti-fade mounting medium containing 4′,6-diamidino-2-phenylindole (DAPI, Invitrogen, Carlsbad, CA, USA, P36962) and imaged using a Zeiss (Oberkochen, Germany) 710 laser scanning confocal microscope equipped with a 63 × objective.

### 4.8. Acute Hippocampal Slice Preparation

Adult male and female (5–6 weeks old) mice were used for all acute slice electrophysiological recordings. Mice were anesthetized with isoflurane and decapitated. Brains were rapidly removed and washed with ice-cold sucrose cutting solution. The sucrose solution was made up of the following (in mM): 60 NaCl, 3 KCl, 28 NaHCO_3_, 1.25 NaH_2_PO_4_, 7.5 Glucose, 0.5 CaCl_2_, 4.5 MgCl_2_. Brain hemispheres were dissected and mounted following a 45° cut of the dorsal cerebral hemisphere(s). Modified transverse slices (300 μm) were made by a Leica (Wetzlar, Germany, VT1200S) vibrating microtome in ice-cold sucrose that was continuously bubbled with carbogen (95% O_2_/5% CO_2_). Slices were recovered at 32 °C in sucrose solution for 15 min, at which time the solution temperature was slowly lowered to room temperature where it remained for the remainder of the recording day. Slices were exposed to pharmacological treatments (treatment or vehicle) during the slicing procedure and in recovery.

### 4.9. Whole-Cell Current Clamp Recordings

Following a 2-h recovery in sucrose cutting solution with or without pharmacological treatment (SB 203580 5 µM and 0.1% DMSO vehicle, respectively), hippocampal slices were transferred to a recording chamber submerged in artificial cerebral spinal fluid (ACSF) with the temperature maintained at 33 °C (±1 °C). The ACSF consisted of the following (in mM): 125 NaCl, 2.5 KCl, 25 NaHCO_3_, 1.25 NaH_2_PO_4_, 25 glucose, 2 CaCl_2_, (pH 7.4). In select recordings, 5 µM SB 203580 was added to the bath solution. The recording chamber was continuously perfused with carbogen-bubbled ACSF at a rate of 3 mL/min. Somatic whole-cell patch clamp recordings were performed on identified somata of hippocampal CA1 pyramidal neurons, which were viewed using infrared differential interference contrast (DIC) on an upright Zeiss (Oberkochen, Germany) Examiner. Cells were patched with 4–5 MΩ borosilicate glass pipettes pulled from a Narishige (Amityville, NY, USA) vertical puller and filled with K^+^ gluconate-based intracellular solution consisting of the following (in mM): 20 KCl, 130 K-gluconate, 2 MgCl, 0.1 EGTA, 2 Na_2_ATP, 0.3 NaGTP, 10 HEPES, 10 Phosphocreatine with pH adjusted with KOH, and HCl to a final value of 7.25–7.30 and an osmolarity of 290–300 mOsm.

AP firing properties were measured from whole-cell recordings in current clamp mode in the conditions described above. All data were recorded with a Multiclamp 700B amplifier (Molecular Devices, San Jose, CA, USA) and a Digidata 1440A digitizer. Signals were low-pass filtered at 5 kHz and digitized at 10 kHz using Clampex 10.7 software and were acquired in bridge balance mode to compensate for series resistance. Liquid junction potential was not corrected for. Passive membrane properties were measured after initial break-in in order to avoid dialysis as a result of solution exchange. Whole-cell capacitance and series resistance were measured from a Multiclamp 700B commander (Molecular Devices, San Jose, CA, USA). A voltage step of −10 mV was used, and the decay tau of the whole-cell capacitive transient current was used to calculate these parameters. Recordings where series resistance exceeded 25 MΩ or resting membrane potential was more depolarized than −55 mV were discarded. Input resistance was calculated as the slope of the current-voltage (I-V) curve in response to current steps from −50 to 50 pA in 50 pA steps (three steps in total). To evoke action potentials in patched CA1 pyramidal neurons, square 500 ms current pulses were elicited in 50 pA steps with current injections ranging from −200 pA to +300 pA. Two sweeps at each magnitude were elicited and the average response of these sweeps was used for each cell. All measures of action potential waveform were taken from the first spike in response to a 150 pA injection and inter-spike measurements, including inter-spike interval, were recorded between the first two spikes in a train elicited by a 150 pA square current. This current magnitude was used as it was the minimum current that provoked AP firing in 100% of cells patched. Additionally, second-long ramp current injections were elicited at 400 pA/sec and the latency to fire was calculated as the time from the initiation of the current injection to AP threshold (first AP). AP threshold and rheobase were measured from ramp injections, with rheobase recorded as the current magnitude required to produce the first AP (at threshold voltage) in the ramp.

### 4.10. Seizure Behavioral Assays

Kainic acid (Sigma, St. Louis, MO, USA, K0250) was administered i.p. at a dose of 25 mg/kg. Animals were monitored for 60 min after the injection. Behavioral responses were recorded using a video camera and scored using the following: stage 0, normal behavior; stage 1, immobility and rigidity; stage 2, head bobbing; stage 3, forelimb clonus and rearing; stage 4, continuous rearing and falling; stage 5, clonic–tonic seizure; stage 6, death (Racine, 1972). Total seizure scores were calculated by summing up every five-minute score. The behavioral assessments described above were performed in a blind manner.

### 4.11. Statistical Analysis

Biochemistry and behavior data were analyzed by Origin 2018b (Northampton, MA, USA) by two-tailed Student’s *t*-test and two-way ANOVA, respectively. Electrophysiological data were analyzed by GraphPad Prism 7 (San Diego, CA, USA, 7.0 d). For all electrophysiological experiments, the experimenter was blinded to the genotypes. For analysis of the pharmacological impact on single AP parameters and in response to ramp current injections in WT and Kv4.2TA slices, a one-way ANOVA (ordinary), or one-way ANOVA on ranks (Kruskal–Wallis test) was used and was corrected for multiple comparisons with Dunnett’s test (ordinary) or Dunn’s test (ranks). The use of parametric or non-parametric analysis was determined after testing for normal distribution of the data using the D’Agostino and Pearson normality test (alpha level = 0.05). Non-parametric statistics were used if the data failed normality testing. For all analysis of firing frequency in response to sequential current steps, a two-way ANOVA with Tukey’s post hoc test was used. All the data are presented as mean ± SEM.

## Figures and Tables

**Table 1:** Passive membrane properties and single action potential (AP) parameters (mean ± SEM).

Parameter	WT	WT (SB 203580)	Kv4.2TA	Kv4.2TA (SB 203580)
RMP (mv)	−60.5 ± 0.86	−60.1 ± 0.72	−58.8 ± 0.62	−58.9 ± 0.60
Whole-cell capacitance (pF)	16.2 ± 1.0	22.4 ± 1.6	17.1 ± 0.77	17.8 ± 1.3
R_input_ (MΩ)	228.8 ± 18.2	273.4 ± 19.7	231.2 ± 13.4	228.9 ± 16.0
Time to AP Peak (ms)	0.84 ± 0.1	1.07 ± 0.1	0.9 ± 0.1	0.89 ± 0.1
AP amplitude (mV)	81.0 ± 3.3	79.6 ± 3.7	76.7 ± 2.8	75.1 ± 3.3
AP half-width (ms)	1.5 ± 0.1	1.7 ± 0.2	1.6 ± 0.1	1.6 ± 0.1
AP threshold (mV)	−40.1 ± 1.1	−35.9 ± 1.5	−34.9 ± 1.4 ^a^	−37.7 ± 1.4

^a^*p* < 0.05.
